# Effect of Bovine Serum Albumin Treatment on the Aging and Activity of Antibodies in Paper Diagnostics

**DOI:** 10.3389/fchem.2018.00161

**Published:** 2018-05-08

**Authors:** Ziwei Huang, Thomas Gengenbach, Junfei Tian, Wei Shen, Gil Garnier

**Affiliations:** ^1^BioPRIA, Department of Chemical Engineering, Monash University, Clayton, MO, Australia; ^2^CSIRO Manufacturing, Clayton, MO, Australia

**Keywords:** antibody bioactivity, BSA, paper diagnostic, protein stability, antibody adsorption

## Abstract

Paper and cellulosic films are used in many designs of low-cost diagnostics such as paper-based blood grouping devices. A major issue limiting their commercialization is the short stability of the functional biomolecules. To address this problem, the effect of relative humidity (RH) and bovine serum albumin (BSA) on the antibody bioactivity and the surface chemical composition of a paper blood typing biodiagnostic were studied. An IgM blood typing antibody was physisorbed from solution onto paper - with or without BSA pretreatment, and aged for periods up to 9 weeks under various conditions with a series of RH. The blood typing efficiency of the antibodies and the substrate surface chemical composition were analyzed by image analysis and X-ray photoelectron spectroscopy (XPS), respectively. This study tests two hypotheses. The first is that the hydroxyl groups in paper promote antibody denaturation on paper; the second hypothesis is that proteins such as BSA can partially block the hydroxyl groups within paper, thus preserving antibody bioactivity. Results show that high RH is detrimental to antibody longevity on paper, while BSA can block hydroxyl groups and prolong antibody longevity by almost an order of magnitude—regardless of humidity. This study opens up new engineering concepts to develop robust and marketable paper diagnostics. The simplest is to store paper and antibody based diagnostics in moisture proof packages.

## Introduction

Paper and cellulosic films have emerged as powerful platforms to engineer biomedical diagnostics for detecting blood glucose, blood groups, and pathogens (Pohanka et al., 2007). However, major obstacles, including sensor instability and poor longevity, have restricted paper biosensors commercialization. For instance, paper-based blood grouping devices containing blood grouping antibodies on paper provide reproducible results for only up to 1 month (Guan et al., 2014). Paper diagnostics also often show poor reproducibility in quantification tested months apart (Delaney et al., 2011). These drawbacks are believed to result from the antibody instability. Antibody denaturation in solution is caused by antibody conformational change from protein unfolding and aggregation (Gombotz et al., 1994). However, the mechanism by which antibodies physisorbed on paper lose their bioactivity is poorly understood. Also unknown is whether paper can be modified to stabilize physisorbed antibodies. Efficient commercialization of paper biosensors requires a robust understanding of the mechanisms behind antibody functionality and stability loss.

While a minimum amount of water is known as essential for antibodies to interact with antigens (Chou and Morr, 1979; Bhat et al., 1994), little is known regarding the functionality of physisorbed antibody under excess water/moisture, especially for paper and hydrophilic cellulosic substrates. Increased relative humidity (RH) was reported as detrimental to antibody longevity using model papers made of eucalyptus fibers (Huang et al., 2017a). Water molecules can diffuse between the hydrogen-bonded fibrils of the cellulose fiber cell wall to decrease the degree of internal bonding, presumably releasing accessible hydroxyl groups (Kaarlo, 2008). These hydroxyl groups may contribute to antibody bioactivity loss through biomolecule immobilization into an unfavorable conformation on paper. Thin papers of high wet strength and regular structure, similar to commercial paper towels (PT), represent performant substrate for paper biosensor, which may act differently to relative humidity (RH). If the hydroxyl surface concentration plays an important role in antibody denaturation on paper, altering their surface concentration on paper would be a convenient strategy to increase antibody longevity and stability.

Using model surfaces, antibody bioactivity loss was reported to involve biomolecule interaction with cellulose surface hydroxyl groups, triggering antibody irreversible adsorption and possible change of conformation (Huang et al., 2017b). This theory has not been studied using paper, especially low-density papers such as paper towels. This exploration is an objective of this study. A second objective is to investigate whether adsorbing a layer of sacrificial protein on paper may prevent antibodies denaturation. Bovine serum albumin (BSA) is a promising protein for paper surface treatment as it renders surfaces inert to protein adhesion (Nakanishi et al., 2004). This study also investigates the effect of BSA as a protective protein layer on paper substrate to increase antibody longevity for blood typing paper diagnostics.

## Materials and methods

### Materials

IgM antibody, Anti-A FFMU (For Further Manufacturing Use), was purchased from Alba Bioscience (United Kingdom). A standard Paper towel (28 g/m^2^) manufactured by Kimberly–Clark professional (Australia) was used as substrate. BSA and phosphate buffered saline (PBS) were purchased from Sigma Aldrich (USA). CSL Revercell™ 15% which contains 15% of RBCs (Group A1 and B cells) was concentrated and used to make 40% red blood cells (RBCs) suspensions in PBS.

### Humidity controlled environment

Based on the definition of salt water activity, saturated salt solutions in air-tight desiccators were used to control the environmental relative humidity (RH). Five different salts were selected to achieve environmental relative humidity (RH) (Table 1) ranging from 6.4 to 84.3 % (Hong et al., 2005). Distilled water was used to maintain the RH at 100%.

**Table 1:** Relative humidity of saturated salt solutions at equilibrium under 20°C (Greenspan, 1977).

**Salt**	**Relative Humidity (%)**
Lithium Bromide	6.4
Magnesium Chloride	32.8
Sodium Bromide	57.6
Potassium Iodide	68.9
Potassium Chloride	84.3
Distilled Water	100

### Sample preparation and antibody bioactivity test

The paper towel was cut into squares (1 cm x 1 cm). A 10 μL droplet of 20% BSA solution was applied on each paper square and air-dried in the laboratory at 23°C for 1 h. The BSA droplet uniformly wets paper. Half of these paper squares was autoclaved for 15 min under 10 psi (around 69 kPa and 121°C) to deactivate the BSA on paper. Alba Anti-A FFMU was diluted in MilliQ water (1:4 v/v), and then a 10 μL droplet of diluted solution was applied to BSA treated paper squares, both air-dried (BSA-PTa), and heated (BSA-PTh) along with untreated paper squares (PT), and air-dried for 1 h before aging. CSL Revercell™ 15% was concentrated into 40% cell solutions and 10 μL droplets of solutions were used for the individual tests. Group A1 cells served as the antigen-positive cells and Group B cells as the antigen-negative cells.

As reported (Huang et al., 2017a), the result of a bioactivity test with the samples prepared on the day served as the reference (100% bioactivity). Relative intensity (*I*_*R*_) was calculated with the following equation:

(1) $$I_{R}=\frac{I_{x}^{+}-I_{x}^{-}}{I_{0}^{+}-I_{0}^{-}}\times 100\%$$

Where $I_{x}^{+}$ and $I_{x}^{-}$ are the color intensity of the stain on aged paper squares on a given test day; $I_{0}^{+}$ and $I_{0}^{-}$ are the color intensity resulting from reference paper samples. $I_{x}^{+}$ and $I_{0}^{+}$ are results from Group A1 cells (antigen-positive); $I_{x}^{-}$ and $I_{0}^{-}$ are results from Group B cells (antigen-negative). On a given test day, three samples were analyzed with A1 cells and one with B cells. All graphs of the antibody longevity study were prepared using GraphPad Prism 6, with “one phase decay” curve fitting. The equation of the model is:

(2) $$Y=(Y_{0}-Plateau)\;*\;exp(-K\;*\;X)+Plateau.$$

Where Y_0_ is the Y value at time zero. It is expressed in the same units as Y. Plateau is the Y value at infinite time, expressed in the same units as Y. K is the rate constant, expressed in reciprocal of the X axis time units.

### Surface property analysis

X-ray photoelectron spectroscopy (XPS) was selected to measure the chemical composition of the paper surface. To simulate the condition of the antibodies in liquid contacting paper surface, BSA-PTa and BSA-PTh samples were rinsed with MilliQ water before analyzing.

XPS analysis was performed using an AXIS Ultra DLD spectrometer (Kratos Analytical Inc., Manchester, UK) with a monochromated Al Kα source at a power of 180 W (15 kV × 12 mA), a hemispherical analyzer operating in the fixed analyzer transmission mode and the standard aperture (analysis area: 0.3 mm × 0.7 mm). The total pressure in the main vacuum chamber during analysis was typically between 10^−9^ and 10^−8^ mbar. Survey spectra were acquired at a pass energy of 160 eV. To obtain detailed information on chemical structure and oxidation states, high resolution spectra were recorded from individual peaks at 20 eV pass energy (yielding a typical peak width for polymers of < 1.0 eV).

Each specimen was analyzed at an emission angle of 0° as measured from the surface normal. Assuming typical values for the electron attenuation length of relevant photoelectrons, the XPS analysis depth (from which 95% of the detected signal originates) ranges between 5 and 10 nm for a flat surface.

For quantification, data processing was performed using CasaXPS processing software version 2.3.15 (Casa Software Ltd., Teignmouth, UK). All elements present were identified from survey spectra. The atomic concentrations of the detected elements were calculated using integral peak intensities and the sensitivity factors supplied by the manufacturer. Binding energies were referenced to the C 1 s peak at 285 eV (aliphatic hydrocarbon). Peak assignments are based on “High Resolution XPS of Organic Polymers, the Scienta ESCA300 Database” (Beamson and Briggs, 1992). The accuracy associated with quantitative XPS is ca. 10–15%.

Precision (i.e., reproducibility) depends on the signal/noise ratio and is usually much better than 5%.

## Results and discussion

**Figure 1:**
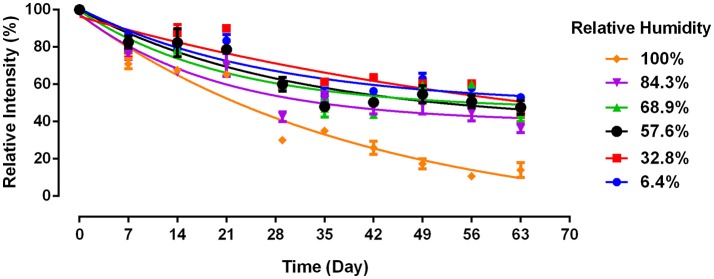
Antibody longevity on paper towel as a function of relative humidity. Antibody A physisorbed on paper towel and aged at 23°C (without BSA). Data is presented as Mean ± SEM (*n* = 3).

The effect of relative humidity (RH) on a commercial paper usable for diagnostics, such as a paper towel (PT), has never been studied and few attempts have been reported to increase antibody longevity or to understand the mechanisms. Here, the effect of RH on antibodies physisorbed on a paper towel was studied under different RH conditions (Figure 1). The relative intensity was calculated following Equation 1 to quantify antibody bioactivity. The paper towel is compatible with blood typing Anti-A IgM Antibody under most humidity conditions. However, high relative humidity (100% RH) accelerates the decay of antibody physisorbed on paper towel.

Antibody bioactivity on paper towel decreases non-linearly with time over 63 days under all RH conditions. At the end of a 2 months aging period, the antibody activity ranges from around 50% of its original level at 6.4% RH, to below 20% at 100% RH. There is no significant difference in loss of antibody activity for aging conditions with RH ranging from 6.4 to 84.3%. However, a significantly lower antibody bioactivity is observed after 1 month and onwards under 100% RH. This confirms previous finding that 100% RH accelerates antibody decay on model paper substrate (Huang et al., 2017a), which however reported a much faster decay. Paper towel differs in composition from the model paper in two aspects. First, it is made of longer softwood fibers instead of the short eucalyptus pulp in the model paper. Chemical compositions of fibers are however similar. Second, PT contains polymeric additives. All paper towels contain a wet strength polymeric agent, typically a cationic polyamideamine-epichlorohydrin (PAE) at 2–5 Kg/T fibers, often a dry strength agent such as an anionic carboxyl methyl cellulose (CMC, at 2–3 Kg/T) and sometimes a cationic surfactant (quaternary amine) used as softeners (at around 1 Kg/T). This combination of additives results in absorbent and strong paper towels under wet and dry conditions, attributes also required for paper diagnostics.

BSA is commonly used as a blocking reagent in bioanalytical assays for its high adsorption rate on both hydrophobic and hydrophilic surfaces (Gibbs and Kennebunk, 2001; Nakanishi et al., 2004). BSA was found to strongly adsorb on hydrophilic surfaces and no desorption occurred after washing with PBS, 1M NaCl, or SDS solution (Jeyachandran et al., 2009). This renders BSA attractive to modify the chemical surface composition of paper. Furthermore, BSA contains significantly less hydroxyl groups than the cellulose of paper. Among the 20 different amino acids composing BSA, Serine (Ser), and Threonine (Thr) are the only two which contain hydroxyl groups. These two amino acids represent around 11% of the total amino acid residues in BSA (32 Ser and 34 Thr out of 607 amino acids, Table 2) (Hirayama et al., 1990).

**Table 2:** Amino acid composition of the 607 amino acids in BSA (Hirayama et al., 1990).

**Ala**	**48**	**Gly**	**17**	**Met**	**5**	**Ser**	**32**
Cys	35	His	16	Asn	14	Thr	34
Asp	41	Ile	15	Pro	28	Val	38
Glu	58	Lys	60	Gln	21	Trp	3
Phe	30	Leu	65	Arg	26	Tyr	21

Table 3 shows the surface chemical composition measured by XPS analysis on paper before (PT) and after applying BSA (BSA-PTa and BSA-PTh). The paper towel surface composition measured is close to the values of cellulose (nil of N/C and 0.83 of O/C; Beamson and Briggs, 1992), except for the presence of a measurable concentration of hydrocarbon (C1). This type of hydrocarbon is probably caused by the polymeric additives and some low adsorption of ambient hydrocarbons, and is commonly observed (Huang et al., 2017a). In contrast, the compositions corresponding to both air-dried BSA-treated PT (BSA-PTa) and heated BSA-treated PT (BSA-PTh) are very different and consistent with the presence of a protein layer on the surface (Coen et al., 2001; Browne et al., 2004). The high concentration of nitrogen (N/C of 0.153 and 0.233, respectively) and sulfur (S/C of 0.006 and 0.010, respectively), and similar values for C 3, corresponding to O-C-O, C = O and N-C = O (C3/C of 0.167 and 0.201, respectively) are typical and reflect the abundance of amides in proteins (peptide bonds linking amino acids).

**Table 3:** Surface chemical composition of three types of paper.

	**Paper Towel (PT)**		**BSA treated PT, air-dried (BSA-PTa)**		**BSA treated PT, Heated (BSA-PTh)**		
	**Mean**	**Dev**.	**Mean**	**Dev**.	**Mean**	**Dev**.	**Chemical functionality**
C 1	0.193	0.002	0.498	0.033	0.517	0.000	C-C, C-H
C 2	0.614	0.000	0.322	0.022	0.262	0.001	C-O, C-N
C 3	0.180	0.001	0.167	0.013	0.201	0.004	O-C-O, C = O, N-C = O
C 4	0.013	0.000	0.014	0.002	0.020	0.005	O-C = O
N	0.013	0.001	0.153	0.007	0.233	0.003	N-C, N-C = O
O	0.608	0.001	0.308	0.008	0.253	0.002	
S	0.001	0.000	0.006	0.000	0.010	0.000	

*Data are presented as atomic ratios X/C, i.e., atomic concentrations of element/species X relative to that of carbon [mean values (+/– deviation) of measurements at two different locations on each sample]*.

From the chemical structure and composition of the amino acids forming BSA (Table 2), ratios of chemical atoms can be calculated. Theoretical nitrogen to carbon atomic ratio (N/C) of 0.266 and sulfur to carbon atomic ratio (S/C) of 0.013 were calculated for BSA. This assumes that the BSA bulk concentration is identical to its surface composition upon physisorption on paper. This calculation was confirmed by Ithurbide's work revealing identical theoretical value of N/C, while no values of S/C or O/C were provided (Ithurbide et al., 2007). The experimental N/C and S/C values of BSA-PTa and BSA-PTh fall between the theoretical value of BSA powder and cellulose fiber, which suggests that some of the cellulosic PT substrate is still detected by XPS. This is either caused by partial BSA surface coverage or by a very thin layer of BSA on paper (<10 nm). BSA coverage on solid surface was reported to vary with both BSA concentration and adsorption time (Jeyachandran et al., 2009). In our study, a very concentrated BSA solution was used (20%). It is likely that BSA coverage on paper is complete, with even the possibility of BSA aggregate formation. The detection of cellulose in XPS analysis probably results from the thin layer of BSA. The three dimensions of a BSA molecule are 14 × 4 × 4 nm (Wright and Thompson, 1975). As XPS can penetrate sample up to 10 nm, this suggests that BSA molecules adsorb on paper prefer a flat orientation and form a layer thinner than 10 nm, thus exposing some cellulose in the layer analyzed. While BSA was denatured by heat (BSA-PTh sample), both the BSA molecular conformation and its layer thickness on paper could change, which may contribute to the minor differences observed between the BSA-PTa and BSA-PTh samples.

**Figure 2:**
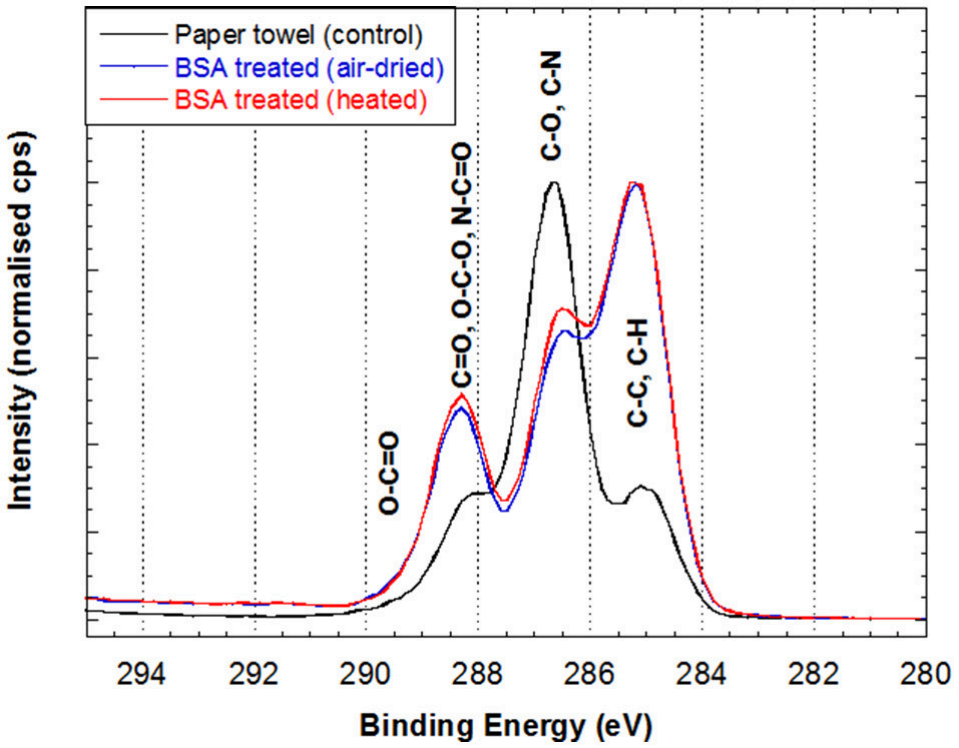
High resolution carbon spectrum of 3 different paper towel samples: (1) original paper towel, (2) air-dried BSA-treated PT (BSA-PTa) and (3) heated BSA-treated PT (BSA-PTh). See text for details.

BSA modifies the paper surface chemical composition, as shown by the significant peak changes in the carbon spectrum after BSA treatment (Figure 2). The peak originating from C-O and C-N based species (including hydroxyls) in both BSA-PTa (0.322) and BSA-PTh (0.262) are less than half of the value in PT (0.614). These results confirm that both BSA treatment air-dried (BSA-PTa) and heated (BSA-PTh) significantly decrease paper surface hydroxyl concentration.

**Figure 3:**
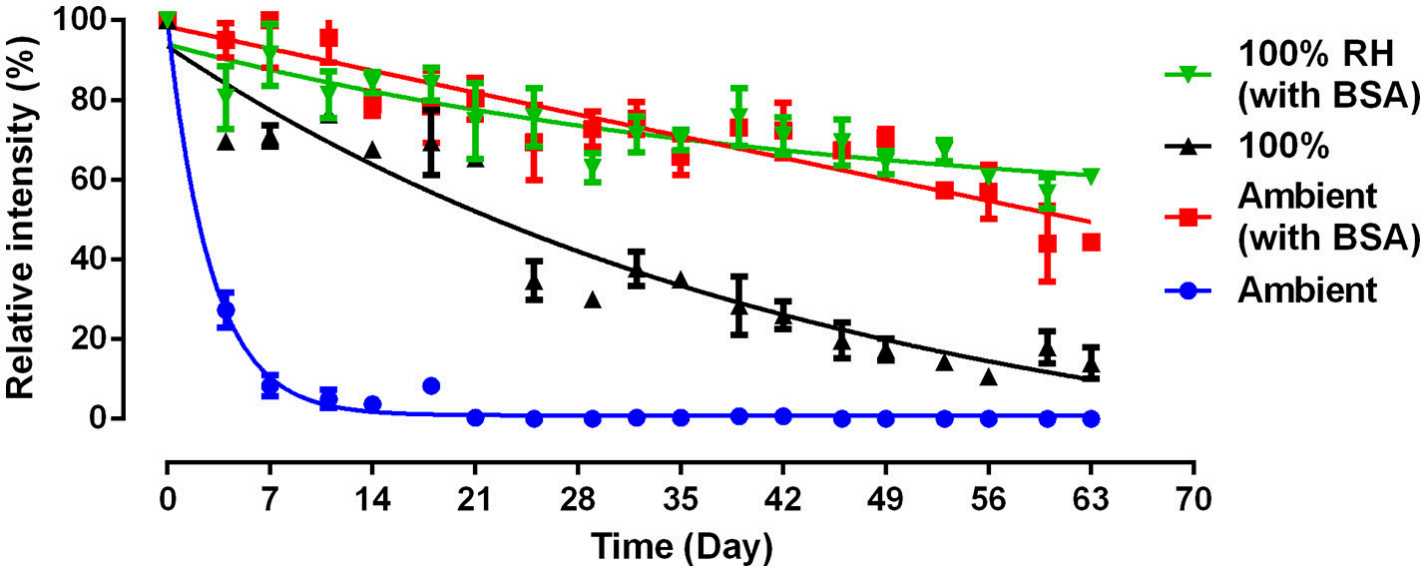
Effect of BSA and humidity on the activity and stability of antibody physisorbed on paper towel. 100% relative humidity (RH): samples were stored at 23°C; Ambient: samples were stored in lab with normally fluctuating temperature and humidity.

We further examined whether BSA treated paper towel (BSA-PTa) can prolong antibody longevity under ambient conditions and for the most detrimental condition (100% RH). PT shows poor ability to prevent antibody decaying with time (Figure 3). Under ambient condition, antibodies on PT exhibit a bioactivity close to 0% on Day 7, while BSA treated PT kept the antibody bioactivity over 50% after 63 days. The antibodies on PT under ambient environment decays faster than PT in 100% RH environment (Figure 3) in this experiment, which differs from Figure 1. This difference may be caused by the fluctuating Australian conditions, both temperature and RH. Temperature impacts paper swelling and antibody denaturation (Vermeer and Norde, 2000), hence affecting antibody bioactivity on PT.

BSA can improve the antibody stability by one order of magnitude (9x, Figure 3). The prolonged antibody longevity with BSA treated paper is probably associated with a more favorable antibody molecular conformation upon adsorption, related to the reduced hydroxyl concentration on the paper surface caused by BSA. BSA prolong antibody bioactivity by 9 times even under the harshest conditions of moisture saturation (100% RH). This highlights the role of the cellulose hydrogen bonding ability in immobilizing antibodies under unfavorable configuration during aging. Adsorbed BSA dried onto paper does not readily nor significantly desorb upon washing with conventional buffer solutions (Jeyachandran et al., 2009). As a result, BSA treated paper remains insensitive to moisture and keeps reducing the amount of free hydroxyl groups under 100% RH to attenuate the detrimental effect of moisture on antibody bioactivity. The stable BSA layer is little affected by RH. These results suggest that the BSA biomolecules permanently alter the chemical composition of the paper surface, reducing the amount of available hydroxyl groups under any environment.

## Conclusion

Anti-A IgM blood typing antibodies were physisorbed from solution onto a paper towel, pretreated or not with BSA, and aged up to 9 weeks under various relative humidity (RH). The antibody bioactivity over time and the chemical composition of the bioactive paper were measured by image analysis and XPS, respectively. Two hypotheses were tested and confirmed in this study. The first is that the hydroxyl groups in paper play an important role in promoting antibody denaturation; the second is that macro-proteins adsorbed onto paper, such as BSA, disrupts the interaction of the hydroxyl groups on paper with the antibody molecules, preserving antibodies functionality on paper.

High relative humidity (100% RH) was found to be the most detrimental condition for denaturing Anti-A IgM blood typing antibodies adsorbed on paper. This condition was selected to accelerate the antibody aging on paper, with and without BSA treatment. Surface chemical composition measured by XPS revealed that BSA treatment reduced by more than half the concentration of surface hydroxyl groups, and the functionality study revealed the BSA treatment to increase antibody longevity by up to 9 times, under both ambient condition and 100% RH, 23°C.

This study suggests that hydroxyl groups are important in antibody bioactivity loss not only on cellulosic films (Huang et al., 2017b) but also on paper and probably any substrate in diagnostics. With BSA treatment, the concentration of surface hydroxyl available is significantly reduced and paper becomes insensitive to environmental moisture. The physisorbed antibodies are more stable on BSA-treated paper as they retain a conformation closer to their original structure in solution. This study provides a new perspective to understand the denaturation mechanism of functional proteins such as antibody and enzyme physisorbed onto surfaces. Paper-based biosensor longevity can now be increased, hopefully driving cost down and facilitating paper–biodiagnostics shelf life and supply chain. Solutions as simple as water vapor resistant packaging for all paper and antibody based diagnostics can make significant differences.

## Author contributions

ZH: provided the idea of using BSA for blocking hydroxyl groups, conducted all antibody longevity experiments, analyzed the data, discussed the XPS findings with TG, and wrote the manuscript; TG: performed XPS surface analyses, discussed the data and wrote the XPS analysis section in the manuscript; JT and WS: provided the idea of testing humidity effects on antibody longevity; GG: provided in-depth guidance regarding data analyzing, as well as reviewed, and revised the manuscript.

### Conflict of interest statement

The authors declare that the research was conducted in the absence of any commercial or financial relationships that could be construed as a potential conflict of interest.

## References

[B1] Beamson G. Briggs D. (1992). High resolution XPS of organic polymers: the Scienta ESCA300 database. Chichester: Wiley.

[B2] Bhat T. N. Bentley G. A. Boulot G. Greene M. I. Tello D. W. Dall'Acqua S.. (1994). Bound water molecules and conformational stabilization help mediate an antigen-antibody association. Proc. Natl. Acad. Sci. U.S.A. 91, 1089–1093. 10.1073/pnas.91.3.1089 8302837 PMC521459

[B3] Browne M. Lubarsky G. Davidson M. Bradley R. (2004). Protein adsorption onto polystyrene surfaces studied by XPS and AFM. Surf. Sci. 553, 155–167. 10.1016/j.susc.2004.01.046

[B4] Chou D. Morr C. (1979). Protein-water interactions and functional properties. J. Am. Oil Chem. Soc. 56, A53–A62.

[B5] Coen M. C. Lehmann R. Gröning P. Bielmann M. Galli C. Schlapbach L. (2001). Adsorption and bioactivity of protein A on silicon surfaces studied by AFM and XPS. J. Coll. Interface Sci. 233, 180–189. 10.1006/jcis.2000.7240 11121264

[B6] Delaney J. L. Hogan C. F. Tian J. Shen W. (2011). Electrogenerated chemiluminescence detection in paper-based microfluidic sensors. Anal. Chem. 83, 1300–1306. 10.1021/ac102392t 21247195

[B7] Gibbs J. Kennebunk M. E. (2001). Effective blocking procedures. ELISA Tech. Bull. 3, 1–6.

[B8] Gombotz W. R. Pankey S. C. Phan D. Drager R. Donaldson K. Antonsen K. P.. (1994). The stabilization of a human IgM monoclonal antibody with poly (vinylpyrrolidone). Pharm. Res. 11, 624–632. 10.1023/A:1018903624373 8058628

[B9] Greenspan L. (1977). Humidity fixed points of binary saturated aqueous solutions. J. Res. Natl. Bur. Stand. 81, 89–96. 10.6028/jres.081A.011

[B10] Guan L. Cao R. Tian J. McLiesh H. Garnier G. Shen W. (2014). A preliminary study on the stabilization of blood typing antibodies sorbed into paper. Cellulose 21, 717–727. 10.1007/s10570-013-0134-x

[B11] Hirayama K. Akashi S. Furuya M. Fukuhara K. I (1990). Rapid confirmation and revision of the primary structure of bovine serum albumin by ESIMS and Frit-FAB LC/MBiochemical, S., and biophysical research communications. Biochem. Biophys. Res. Commun. 173, 639–646. 10.1016/S0006-291X(05)80083-X 2260975

[B12] Hong T. D. Edgington S. Ellis R. H. de Muro M. A. Moore D. (2005). Saturated salt solutions for humidity control and the survival of dry powder and oil formulations of *Beauveria bassiana* conidia. J. Inverteb. Pathol. 89, 136–143. 10.1016/j.jip.2005.03.007 15876439

[B13] Huang Z. Gengenbach T. Tian J. Shen W. Garnier G. (2017a). The role of polyaminoamide-epichlorohydrin (PAE) on antibody longevity in bioactive paper. Coll. Surf B Biointerfaces 158:197–202. 10.1016/j.colsurfb.2017.07.005 28692875

[B14] Huang Z. Raghuwanshi V. S. Garnier G. (2017b). Functionality of immunoglobulin g and immunoglobulin m antibody physisorbed on cellulosic Films. Front. Bioeng. Biotechnol. 5:41. 10.3389/fbioe.2017.00041 28770196 PMC5511829

[B15] Ithurbide A. Frateur I. Galtayries A. Marcus P. (2007). XPS and flow-cell EQCM study of albumin adsorption on passivated chromium surfaces: influence of potential and pH. Electrochimica Acta 53, 1336–1345. 10.1016/j.electacta.2007.04.109

[B16] Jeyachandran Y. L. Mielczarski E. Rai B. Mielczarski J. A. (2009). Quantitative and qualitative evaluation of adsorption/desorption of bovine serum albumin on hydrophilic and hydrophobic surfaces. Langmuir 25, 11614–11620. 10.1021/la901453a 19788219

[B17] Kaarlo N. (Ed.). (2008). Paper Physics, Paperi ja Puu Oy.

[B18] Nakanishi J. Kikuchi Y. Takarada T. Nakayama H. Yamaguchi K. Maeda M. (2004). Photoactivation of a substrate for cell adhesion under standard fluorescence microscopes. J. Am. Chem. Soc. 126, 16314–16315. 10.1021/ja044684c 15600320

[B19] Pohanka M. Skládal P. Kroèa M. (2007). Biosensors for biological warfare agent detection (review paper). Defence Sci. J. 57, 185–193. 10.14429/dsj.57.1760

[B20] Vermeer A. W. Norde W. (2000). The thermal stability of immunoglobulin: unfolding and aggregation of a multi-domain protein. Biophys. J. 78, 394–404. 10.1016/S0006-3495(00)76602-1 10620303 PMC1300647

[B21] Wright A. K. Thompson M. R. (1975). Hydrodynamic structure of bovine serum albumin determined by transient electric birefringence. Biophys. J. 15(2 Pt 1), 137–141. 1167468 10.1016/s0006-3495(75)85797-3 PMC1334600

